# Creating patient-centered health care systems to improve outcomes and reduce disparities

**DOI:** 10.1186/s13584-015-0039-2

**Published:** 2015-08-18

**Authors:** Julie A. Schmittdiel

**Affiliations:** Kaiser Permanente Northern California, Division of Research, 2000 Broadway, Oakland, CA 94612 USA

**Keywords:** Patient-centered care, Disparities, Patient preferences

## Abstract

Health care delivery systems that are designed to understand and meet patient preferences for care have the potential to improve health outcomes and reduce disparities. Studies that rigorously assess patient care preferences in minority and underserved populations, stakeholder engagement, and policies that promote a diverse health care workforce that can address patient preferences are important levers for improving care for vulnerable populations.

## Commentary

In a recent Israel Journal of Health Policy Research article, Amer-Alsheik and colleagues describe the results from their assessment of Israeli Druze women’s preferences when choosing obstetricians and gynecologists [1]. Their cross-sectional study used an anonymous survey of almost 200 women from the Israel Druze community to find that while most women who responded to the survey did not have a preference for the sex of their family physician, almost two-thirds of respondents (63.8 %) preferred to have a female obstetrician/gynecologist. Preference for a female obstetrician/gynecologist was associated with older age and with being more religious (as opposed to self-identifying as being secular), and with having a current obstetrician/gynecologist who was female. Preferring a female obstetrician/gynecologist was also associated with placing less weight on physician professional knowledge as a factor in selecting a provider. While the sample size of the survey is relatively small, and the results confirm the findings of prior studies that have seen a preference for female obstetrician/gynecologists [2], this new study makes important contributions to our understanding of patient preferences for care.

Cultural competency in delivering health care has been recognized by the U.S. National Institutes of Health (NIH) as an essential component for delivering care that meets the needs of diverse patients. A patient’s culture includes both religious and ethnic identity, and can strongly influence beliefs regarding health and wellness. Providers and health care systems that demonstrate cultural competency in understanding and addressing these beliefs are well-positioned to improve access and health care outcomes, and to potentially reduce disparities [3]. The Druze are a part of the Arab minority in Israel, and are recognized as a distinct ethnic and religious community [1]. A relatively small proportion of the Druze (6–7 %) live in Israel, with most of the Druze living in Syria and Lebanon. While Druze religious law grants women significant rights and social status, the women in Druze communities are often governed by traditions that strictly define women’s modesty and restrict their contact with men who are not immediate family members. Previous research has documented that this has sometimes resulted in Druze women delaying or avoiding medical care in order to avoid contact with male physicians, particularly male obstetrician/gynecologists. However, the societal norms for Druze women have undergone significant transformation in recent years, in parallel with striking changes in the role of women across many cultures internationally. Studies such as the one conducted by Amer-Alshiek and colleagues are critical because they seek to assess the current health care preferences among women in a community who may face culturally-specific barriers accessing health care, providing policy-makers with important data for improving the cultural competency of care for a potentially vulnerable population. Further research should continue to assess the needs of minority populations within and outside of Israel in an effort to inform the design of health care systems that seek to improve care for minority populations and ameliorate disparities in care.

Cultural beliefs regarding health care also extend to beliefs on how health care is delivered. Studies across many nations have found that women consistently report preferring a female obstetrician/gynecologist [1, 2], suggesting that a health care system that is culturally competent should be able to meet the demand for female physicians to provide obstetric and gynecologic care. While women make up an increasing proportion of physicians internationally, the physician workforce is critically lacking in diversity, with significant underrepresentation of minorities in both clinical practice and in graduate medical education programs. A recent study in this journal found that the Arab minority in Israel is significantly underrepresented among physicians, and that this is largely due to the underrepresentation of Arab women [4]. Studies have consistently shown that both the gender and the ethnic characteristics of the medical workforce influence patient outcomes, and that gender concordance and race/ethnicity concordance between providers and their patients can both influence outcomes as well [4, 5]. Increasing the diversity of the health care workforce is therefore widely seen as a critical policy lever for improving care and reducing disparities, especially since doctors from underrepresented minorities are often more likely to work in clinical settings that serve minority and vulnerable populations. Israel introduced financial incentives to encourage physicians to work in peripheral regions of the country (which often have high concentrations of minority and vulnerable populations) in 2011, and recent years have seen substantial growth in the number of both Jewish and Arab female physicians in Israel [6]. Addressing the underrepresentation of minorities, particularly minority women, in health care should continue to be a key health policy priority for those seeking to meet the needs of patients who wish to receive care from female providers within their communities.

Creating health care systems that are culturally competent for diverse patient populations, and medical education systems that recruit and retain underrepresented minorities, will require policy initiatives aimed at the health care system-level. Research that will further the goal of improving health care systems’ capacity to both assess patient preferences and meaningfully address those preferences will require stakeholder engagement at the patient, provider, and health care system level [7]. The creation of the Patient Centered Outcomes Research Institute (PCORI) in the United States underscores the importance of including patients as key stakeholders in all aspects of research, and health services and policy researchers are currently seeking to create sustainable approaches to patient stakeholder engagement that are evidence-based [8]. These approaches include patient surveys to help identify patient priorities, and also include working with patient stakeholders to incorporate these priorities and needs into research questions and policy approaches [8]. Work such as that by Amer-Alsheik and colleagues represents a first step in stakeholder-engaged research for religious communities such as the Druze; future research should continue to engage patients and providers in underrepresented communities as part of a larger research agenda to improve the level of culturally-appropriate care.

## Abbreviations

PCORI: Patient Centered Outcomes Research Institute

## References

[1] Amer-Alsheik Q, Alshiek T, Levy JA, Azem F, Amit A, Amir H (2015). Israeli Druze women’s sex preferences when choosing obstetricians and gynecologists. Isr J of Health Policy Res.

[2] Schmittdiel J, Selby JV, Grumbach K, Quesenberry CP (1999). Women’s provider preferences for basic gynecology care in a large health maintenance organization. J Womens Health Gend Based Med.

[3] National Institutes of Health. Clear Communication: Cultural Competency. http://www.nih.gov/clearcommunication/culturalcompetency.htm. Accessed June 30, 2015.

[4] Keshet Y, Popper-Giveon A, Liberman I (2015). Intersectionality and underrepresentation among health care workforce: the case of Arab physicians in Israel. Isr J of Health Policy Res.

[5] Traylor AH, Schmittdiel JA, Uratsu CS, Mangione CM, Subramanian U (2010). The Predictors of Patient-Physician Race and Ethnic Concordance: A Medical Facility Fixed-Effects Approach. Health Serv Res.

[6] Haklai Z, Applbaum Y, Tal O, Aburbeh M, Goldberger NF (2013). Female physicians:trends and likely impacts on healthcare in Israel. Isr J Health Policy Res.

[7] Schmittdiel J, Grumbach K, Selby JV (2010). Health Care System-Based Participatory Research: An approach for sustainable translational research and quality improvement. Ann Fam Med.

[8] Schmittdiel JA, Desai J, Schroeder E, Paolino A, Nichols G, Lawrence J, O’Connor P, Ohnsorg K, Newton K, Steiner J (2015). Methods for engaging stakeholders in comparative effectiveness research: A patient-centered approach to improving diabetes care. Healthc(Amst.).

